# The role of microRNAs involved in the disorder of blood–brain barrier in the pathogenesis of multiple sclerosis

**DOI:** 10.3389/fimmu.2023.1281567

**Published:** 2023-12-14

**Authors:** Asieh Emami Nejad, Seyed Mostafa Mostafavi Zadeh, Hamid Nickho, Ali Sadoogh Abbasian, Azim Forouzan, Mojtaba Ahmadlou, Reza Nedaeinia, Saham Shaverdi, Mostafa Manian

**Affiliations:** ^1^ Department of Biology, Payame Noor University (PNU), Tehran, Iran; ^2^ Department of Molecular Medicine, Faculty of Advanced Technologies in Medicine, Iran University of Medical Sciences, Tehran, Iran; ^3^ Oncopathology Research Center, Iran University of Medical Sciences, Tehran, Iran; ^4^ Department of Immunology, Faculty of Medicine, Iran University of Medical Sciences, Tehran, Iran; ^5^ Department of Internal Medicine, School of Medicine, Amiralmomenin Hospital, Arak University of Medical Sciences, Arak, Iran; ^6^ Department of Biostatistics, School of Medicine, Arak University of Medical Sciences, Arak, Iran; ^7^ Pediatric Inherited Diseases Research Center, Research Institute for Primordial Prevention of Non-Communicable Disease, Isfahan University of Medical Sciences, Isfahan, Iran; ^8^ Department of Medical Laboratory Science, Faculty of Medicine, Islamic Azad University, Kermanshah, Iran; ^9^ Neurosciences Research Center, Isfahan University of Medical Sciences, Isfahan, Iran

**Keywords:** miRNAs, autoimmune, multiple sclerosis, exosomes, therapeutic advances

## Abstract

miRNAs are involved in various vital processes, including cell growth, development, apoptosis, cellular differentiation, and pathological cellular activities. Circulating miRNAs can be detected in various body fluids including serum, plasma, saliva, and urine. It is worth mentioning that miRNAs remain stable in the circulation in biological fluids and are released from membrane-bound vesicles called exosomes, which protect them from RNase activity. It has been shown that miRNAs regulate blood-brain barrier integrity by targeting both tight junction and adherens junction molecules and can also influence the expression of inflammatory cytokines. Some recent studies have examined the impact of certain commonly used drugs in Multiple Sclerosis on miRNA levels. In this review, we will focus on the recent findings on the role of miRNAs in multiple sclerosis, including their role in the cause of MS and molecular mechanisms of the disease, utilizing miRNAs as diagnostic and clinical biomarkers, using miRNAs as a therapeutic modality or target for Multiple Sclerosis and drug responses in patients, elucidating their importance as prognosticators of disease progression, and highlighting their potential as a future treatment for MS.

## 1 Introduction

The American National MS Association of America describes multiple sclerosis as a disease characterized by inflammation that affects myelin sheaths on nerve cells in the spinal cord and brain. Approximately 70% of patients have severe and painful spasms, especially in the legs. Ninety percent of patients with MS complain of sexual problems, which can be caused by the disease or because of the side effects of drugs prescribed to treat this disease (1).

The signs of MS usually occur in two different patterns: A) Periods of sudden exacerbation: these symptoms last from several days to several weeks and recurrence or deterioration in the course of the disease is called an attack or sudden exacerbation, and then the disease goes into remission. This pattern comprises 85% of cases. B) A gradual worsening (progressive form) over time without recovery periods, which comprise 10-15% of cases. In some cases, a combination of these patterns may occur or people may experience periods of relapse and recovery, which later become the progressive form. Loss of BBB integrity leads to the entry of plasma proteins, toxins, and immune cells from the bloodstream into the brain. This influx triggers encephalitis, subsequently contributing to the onset or exacerbation of various neurological disorders and cerebral vascular diseases, including reperfusion/ischemia stroke, hypertension, amyotrophic lateral sclerosis, cerebral cavernous malformation, depression, and multiple sclerosis (2–5).

## 2 Diagnostic measures of MS disease

MS may affect short-term memory rather than long-term memory, attention and concentration, verbal ability, and information processing power. The characteristics of MS are Uhthoff’s phenomenon, which is the severity of the symptoms of the disease when the body temperature rises during physical activity, fever, or hot weather, and Lhermitte’s sign, which is an electric shock-like sensation that occurs on flexion of the neck (1).

Clinical symptoms include:

1. Sensory disorders: numbness of the limbs and local burning feeling in the limbs.
2. Movement disorders: swallowing and speech disorders, weakness and fatigue or muscle spasms, and paralysis of the limbs on one or two sides.
3. Visual disorders: blurred vision, visible field defects, diplopia, and nystagmus (clinical symptoms are primarily unilateral and occur in 60% of cases).
4. Cerebellar disorders: imbalance and tremors of eyes and limbs and dizziness.
5. Involvement of the urinary system: urination disorders and a degree of impotence.
6. Psychological symptoms, cognitive disorders, and depression.

Altered expression of some miRNAs is associated with pathological conditions; the amount of miRNA in the blood or brain of MS patients correlates with the stage of the disease and the progression of the disability. It may also provide timely diagnosis and effective treatment in the future.

There is evidence that apoptosis is an essential homeostatic process responsible for the termination of imbalanced controlled immune responses in MS patients. Several studies have shown that peripheral immune cells of MS patients are relatively resistant to apoptosis under various experimental conditions in comparison with healthy individuals (6, 7).

These and other findings suggest that the induction of peripheral immune cell apoptosis is a potential therapeutic target in MS (8). Gniadek et al. have investigated and found that the immunoregulatory activity of interferon-beta (IFN-β), an important drug for inducing remission in MS, is associated with increased apoptosis.

It has been reported that the most significant changes in miRNA and mRNA expressions in IFN-β-1b occur 1 month after starting treatment. It has been observed that the induction of IFN-β-responsive genes is associated with parallel down-regulation of miRNA expression. It has been shown that the regulation of miRNAs plays a role in the function of molecular mechanisms of IFN-β responses and the treatment of MS.

Studies have shown that most interactions between the expression of hsa-miR-16-5p and hsa-miR-532-5p are associated with IFN-response genes and high levels of the hsa-miR-16-5p expression are in the different blood cell types, especially monocytes. However, the complex interaction between TFs, miRNAs, and genes is still a big challenge. Functionally, some miRNAs (e.g., miR-29 family members) are related to apoptosis and IFN signaling feedback loops. In order to monitor the biological response to treatment and predict individual disease activity and progression, miRNA expression in blood cells may provide a biomarker. It may also be helpful to better comprehend the pathogenic mechanisms and optimize MS treatment (9).

### 2.1 Disturbance in apoptosis of T cells in MS pathogenesis

Seidi et al. have shown that apoptosis is disturbed and dysregulated in T cells of MS patients; also, the expression level of FLIP and Bcl-2 proteins increases compared to the control group, making T cells resistant to apoptosis and remain self-reactive (10). The presence of miRNA profiles in naive T cells has been reported. Investigation of T cell sub-types revealed miRNAs via mechanisms induce their differentiation into pro-inflammatory phenotype in MS patients (11).

There is growing evidence that T and B lymphocytes play central core roles in the immunopathology of MS. During treatment with IFN-β, one of the first lines of disease-modifying drugs used to treat relapsing-remitting MS, researchers have seen a significant reduction in the circulating B-cell CD27 sub-population. Memory T and B cells are considered a subset of cells that are important to MS disease and have been targeted to be eliminated (12). IFN-β treatment reduces the expression of the LMP2A gene of EBV in RRMS patients. Since IFN-β targets the reservoirs of EBV infection, memory B cells indirectly reduce the level of EBV gene expression in MS patients. In addition, in terms of etiopathogenesis of MS, IFN-β induced a decrease in the memory T cell, leading to a reduction in the expression of the EBV2A latent membrane protein gene in treated patients (13).

Rizzo et al. found that IFN-β therapy *in vivo* specifically and significantly induced apoptosis in memory B cells, a substantial increase in apoptotic markers such as Caspase 3 and Annexin-V, through a mechanism requiring FAS-receptor/TACI signaling (Transmembrane activator and CAML interactor) (14–17).

It has also been shown that the glatiramer acetate induces a significant increase in apoptosis in CD4 + T cells *in vitro*. Maddalena Ruggieri et al. have expressed that Glatiramer Acetate induces pro-apoptotic mechanisms through Cyt-c, Bax, and Bcl-2 in the peripheral lymphocytes of MS patients (18). The critical side effects of the drugs include flu-like symptoms, headache, depression, leukopenia, liver problems, thyroid dysfunction, and injection site reactions (19). The expression level of miRNAs such as miR-21 during the response to traumatic brain injury (TBI) suggests that they may affect the pathophysiological process in brain injury. Ge et al. have reported that miR-21 induces angiogenesis and inhibits apoptosis. Studies showed the essential role of T cells and autoantibodies in the pathogenesis of MS, and these autoantibodies have been suggested as diagnostic biomarkers (20). Differential pathogenesis patterns are defined as a result of demyelination in MS lesions:

Type I: It caused the destruction of myelin by T cells, especially TH17, and the activation of macrophages.
Type II: Particular antibodies and complement activation cause demyelination.
Type III: It is related to distal oligodendropathy, and the degenerative changes occur distally, followed by apoptosis.
Type IV: After this, secondary demyelination occurs and leads to primary oligodendrocyte damage (21).

## 3 microRNA

Small non-coding RNAs are 18-25 nucleotides in size and play an important role in various biological processes (22–24). It has been proposed that miRNAs regulate the expression of one-third of human genes (25). The regulation of several mRNAs by one miRNA has high-potential sufficiency because of a deficiency in complete pairing between the miRNA and the 3’UTR-end sequence of the target mRNA. Recent research has shown that coupling six to seven nucleotides of a miRNA to the 3’ region (UTR) of the target mRNA is necessary to detect the target mRNA. In MS, the profile of miRNAs of CNS lesions and the immune system is altered and can affect gene expression in many cell types involved in MS. The investigation of miRNA dysregulation has provided insight into MS pathophysiology and led to alternative methods for earlier diagnosis and treatment of the disease (26). miRNAs affect the development and regulation of the acquired immune system and T and B biological activities (27).

So far, several studies have been developed on the expression of miRNA in CNS patients’ tissue, animal models, inflammatory lesions of other autoimmune diseases, and paraneoplastic diseases. The analysis of miRNAs’ expression in the active, inactive, and regular white matter lesions CNS’ tissue provided a pattern of 50 miRNAs up-regulated and 30 miRNAs down-regulated in MS compared to healthy subjects (28). Laser capture micro-dissection of active lesions showed that most of the increased expression of miRNAs is expressed by B cells, T cells, astrocytes, and macrophages (28).

The significant role of miRNA in disorders is determined by the exchange of disease-related miRNA levels and individual profiles in their immune cell subsets. *In vivo*, using miRNA mimics and inhibitors has been shown to cause miR-21 overexpression related to an increase in the number of TH17 cells and disease severity in the experimental autoimmune disease encephalomyelitis (EAE). Also, the reduction of miR-21 expression and fewer Th17 cells are directly related to mild EAE disease (29). Irregular miRNAs’ expression is associated with CNS disorders, and in particular, some miRNAs are essential for neurogenesis regulation, neuron growth, oligodendrocyte differentiation, and myelin maintenance (30).

In summary, dysregulation of miRNAs in MS lesions contributes significantly to the mechanisms of MS inflammation. It is also clear that modifications in the pattern of miRNAs reflect both the demyelination process and an effort to remyelinate degenerated axons in the CNS. The profile of miRNA expression of blood cells and CNS lesions in MS patients showed its dysregulation, which is related to the pathological disease aspect. Then the disruption of miRNA expression is the background of biochemical modifications in MS. Whether this abnormal expression of miRNAs leads to the onset of this disease is not yet clear. The dysregulation of miRNAs may have a crucial role in various biological processes, including proliferation, development, differentiation, metabolism, apoptosis, angiogenesis, inflammation, and immunity (31, 32).

Multiple sclerosis (MS) is an autoimmune disease where T cells react to self-antigens found in the brain, particularly myelin antigen, which plays a significant role. Regev et al. showed that serum miRNAs could be used as potential biomarkers to diagnose and investigate disease status in MS (33). MRI is often used to demonstrate and investigate MS. It is essential to find blood biomarkers in MS that can aid in diagnosing the disease, determining disease stages, and establishing connections to disability accumulation. Besides cells, miRNAs have been found in various body fluids that are highly stable due to their resistance to ribonucleases. Circulating miRNAs remain stable even after prolonged storage, freeze-thaw cycles, and exposure to extreme pH conditions. Because of their stability and the development of sensitive detection methods for circulating miRNAs, they are excellent candidates for biomarkers. Researchers have observed changes in circulating miRNAs in several diseases (34, 35).

miRNAs are stable in human tissue and blood samples. Therefore, they can play a role as diagnostic or prognostic biomarkers (36). The reaction between miRNA and target's mRNAs is influenced by miRNA expression levels and affected by the presence of SNPs in miRNA genes and miRNA binding sites, which are generally present in UTR of mRNAs-′3 and create miRNA: mRNA pairs. miRNA binding to mRNA is essential to regulate the amount of mRNA and protein expression. Nucleotides 2-7 from the “seed” region are important for the relative complementarity of miRNA and the detection of the target mRNA to identify the mRNA target site. The presence of SNPs in these locations may disrupt or weaken a miRNA’s target or produce an entirely new sequence in the seed region of miRNA that does not bind to any specific mRNA. The diversity of SNPs can increase or decrease the binding strength of miRNAs effective in protein translation; for example, SNPs associated with miRNAs may cause cancer due to inhibiting the function of tumor suppressor miRNAs (37).

### 3.1 The ability to use miRNAs in the diagnosis of diseases

Several studies have been conducted on the profile of miRNAs in two groups of MS patients and control subjects in whole blood, peripheral blood mononuclear cells (PBMC), and brain lesions. All studies have shown that the expression profile of miRNAs is different in MS patients compared to healthy and control subjects. Studies have shown that the expression of miR-155 and miR-326 significantly increased in active brain lesions of MS patients compared to healthy individuals (38). **Table 1** shows the variation of miRNAs’ pattern in Multiple Sclerosis patients.

**Table 1:** Difference of miRNAs pattern in Multiple Sclerosis patients(**UpregulatedmiRNAs,*Down regulated miRNAs).

	Year	Sample	Tissue	Method	DysregulatedmiRNAs
Du et al. (29)	2009	42RR MS( relapsing remitting multiple sclerosis), 42 healthy	PBL, CD4+T cells	qPCR	miR-326
Junker et al. (28)	2009	20 MS; 9healthy	Active and inactive MS lesions	qPCR and Array	miR-650,miR-155, miR-326, miR-142-3p, miR-146a, miR-146b, miR-34a, miR-21, miR-23a, miR-199a, miR-27a, miR-142-5p, miR-193a, miR-15a, miR-200c, miR-130a, miR-223, miR-22, miR-320, miR-214, miR-656, miR-184, miR-139, miR-23b, miR-328, miR-487b, miR-181c, miR-340
Otaegui et al. (39)	2009	9RR MS, 8 healthy controls	PBMCs	qPCR	miR-18b, miR-493, miR-599
Keller et al. (40)	2009	CIS (n = 25) or RRMS (n = 25) and 50 healthy controls	Whole blood	Microarray and Real Time Analyzer	hsa-miR-145 hsa-miR-186 hsa-miR-664 hsa-miR-20b hsa-miR-422a hsa-miR-142-3p hsa-miR-584 hsa-miR-223 hsa-miR-1275 and hsa-miR-491-5p
Lindberg et al. (41)	2010	10RR MS, 10 healthy controls	CD4+ T cells, CD8+ Tcells, B cells	TaqMan Array	miR-485-3p, miR-376a, miR-1, miR-497, miR-193a, miR-200b, miR-126, miR-486, miR-17-5p, miR-34a
De Santis et al. (42)	2010	14RR MS, 14 healthy controls	CD4+CD25+ T cells	microarray	miR-29c, miR-107, miR-21, let-7i, miR-15a, miR-19a, miR-19b, miR-138-2, miR-324-3p, miR-301a, miR-338-5p, miR-22, miR-512-3p, miR-564, miR-886-3p, miR-106b, miR-29a, miR-93, miR-489, miR-148a, miR-590-5p, miR-223, miR-221
Cox et al. (43)	2010	59 treatment naïve MS patients and 37 controls.	Whole blood	RT-PCR	miR-17 miR-20a
Fenoglio et al. (44)	2011	29 MS patients and 19 controls.	PBMCs	qPCR	miR-21, miR-146a, miR-146b
Guerau-de –Arellano et al. (11)	2011	14RR MS, SP MS, or PP MS; 16 healthy controls	Naive CD4+ T cells	TaqMan Array	miR-660, miR-518d-3p, miR-586, miR-128, miR-564, miR-708, miR-378, miR-346, miR-645, miR-566
Waschbisch et al. (45)	2011	74 RRMS and 32 healthy controls	PBMCs	qPCR	miR-19a, miR-21, miR-142-3p, miR-145, miR-146a, miR-146b, miR-155, miR-200c, miR-210, miR-326, miR-629, let-7f
Siegel et al. (46)	2012	5 MS, 4 healthy controls	Plasma	microarray	miR-614, miR-572, miR-1979, miR-648, miR-422a, miR-1826, miR-22
Noorbakhsh F et al. (47)	2011	16 MS patients and 10 controls	Brain tissue	microarray and PCR	(*miR-7, *miR-299-5p, *miR-135a, *miR-218, *miR-129-3p, *miR-9, *miR-128, *miR-130a, *miR-126, *miR-335, *miR-98) (**miR-25, **miR-505, **miR-320b, **miR-320a, **miR-338-3p, **miR-181b, **miR-92a, **mir-155, **miR-584, **miR-219-2-3p, **miR-338-5p, **miR-219-5p, **miR-142-5p)
Sievers C et al. (48)	2012	10 untreated and 10 natalizumab treated RRMS patients , and 10 healthy	B cells	real-time RT-PCR	miR-19b, miR-551a, miR-106b, miR-191, miR-25
MartinelliBoneschi et al. (49)	2012	19 MS; 14 healthy	PBMCs	microarray	let-7g, miR-150
Haghikia et al. (50)	2012	12RR MS, SP MS, or PP MS, 39 patients with other neurological disease	CSF	Microarray and qPCR	miR-922, miR-181c, miR-633
Yang D et al. (51)	2013	40 MS patients and 40 controls	PBMCs	real-time PCR	miR-150, miR-152, miR-181c, miR-197, miR-328, miR-363, let-7g
Gandhi R et al. (52)	2013	10 RRMS , 9 SPMS , and 9 healthy controls	Plasma	qPCR	miR-22, miR-155, miR-210, let-7f
Sondergaard et al. (53)	2013	12 treatment-naïve RRMS and 20 HCs	PBMC	Microarray,real-time qPCR	let-7d, miR-744, and miR-145
Andreas Keller et al. (54)	2013	treatment-naïve patients with CIS (n = 25) or RRMS (n = 25) and 50 healthy controls	Wholeblood	NGS, microarray analysis, and (qRT-PCR).	(**miR-16-2-3p) or (*miR-20a-5p, *miR-7-1-3p)
Magdalena JustynaKacperska et al. (55)	2014	20 patients undergoing relapse and 17 in remission,30 healthy	Wholeblood	qPCR	miR-let-7a, miR-92a, and miR-648a)
Roberta Mancuso et al. (56)	2015	16 primary progressive (PP), 15 secondary progressive (SP), 31 relapsing remitting (RR) MS patients and 15 HCs	Plasma	qPCR	miR-572
Sherry Freiesleben et al. (57)	2016	22 MS patients	Wholeblood	Microarray	let-7b and miR-345
Julia Vistbakka et al. (58)	2016	(62 MS and 21 controls)	Wholeblood	qPCR.	miR-191-5p miR-376c-3p miR-128-3p and miR-24-3p
Saeideh Ebrahimkhani et al. (59)	2017	MS patients (n = 25) and matched healthy controls (n = 11)	Serum exosomal	next generation sequencing	relapsing-remitting MS (RRMS) (miR-15b-5p, miR-451a, miR-30b-5p, miR-342-3p) and progressive MS patient sera (miR-127-3p, miR-370-3p, miR-409-3p, miR-432-5p)
Maria Liguori et al.	2019	19 pediatric MS and 20 pediatric controls (PCs)	Whole blood	NGS, microarray analysis, and (qRT-PCR).	(**let-7a-5p, **let-7b-5p, **miR-25–3p, **miR-125a-5p, **miR-942–5p, **miR-221–3p, **miR-652–3p, **miR-182–5p, **miR-185–5p, **miR-181a-5p, **miR-320a, **miR-99b-5p) (*miR-148b-3p)
NicolettaNuzziello et al. (60)	2018	Adult-Onset Multiple Sclerosis (n=58) healthy controls (n=20)	Whole blood	qPCR, microarray, NGS	miR-320a, miR-125a-5p, miR-652-3p, miR-185-5p, miR-942-5p, miR-25-3p)
Qinghe Yang et al. (61)	2017	79 MS and 58 normal control samples at datasets microarray	Whole blood	microarray analysis and qRT-PCR	miR-30a, miR-93, miR-20b, and miR-20a
María Muñoz-San Martín et al. (62)	2019	46 patients with MS	CSF	TaqMan assays and qPCR	miR-21 and miR-146a/b

### 3.2 The role of MicroRNAs in the expansion and activity of immune cells

miRNAs regulate both innate and adaptive immune responses. miRNAs are expressed with a specific pattern in the immune system cells and play an essential role in their expansion, mutation, and activity (19, 63). In several respects, miRNAs control the activity and development of immune system cells and the differentiation and activation of B and T lymphocytes (64, 65). They also play a role in the pathogenesis of MS. The study by Du et al. on the role of miRNAs in T cells and MS disease showed that miR-326 is related to the helper (CD4+ cells) Th17-producing interleukin-17 (IL-17), which plays a remarkable role in autoimmune diseases such as MS (66). They showed that miR-326 has increased in Th-17 cells of RRMS patients related to the extent of the disease, the differentiation of Th-17 cells, and the inhibition of Ets-1, which is a negative regulator of the differentiation of Th-17 cells (29).

Real-time PCR analysis showed increased expression of miR-145 in PBMC of MS patients (67). In addition, miR-326, miR-155, miR-146a, and miR-142-3p are elevated in the PBMC of MS patients. Another study showed that miR-146a, miR-21, miR-326, and miR-146b increased in PBMC of RRMS patients compared to control subjects (44).

An investigation of T cell subtypes has shown that miRNAs induce their differentiation to pro-inflammatory phenotype in MS patients by several mechanisms (11). It has been shown that miR-16-1 and miR-15a increased in CD4+ T cells in the peripheral blood of RRMS patients. miR-128, miR-27b, and miR-340 have also increased expression in blood T cells of MS patients (68, 69).

miRNA plays a crucial role in the regulation of stem cell self-renewal and differentiation by suppressing specific mRNA in stem cells, leading to their differentiation into daughter cells. This function has been observed in various somatic tissue stem cells, germline stem cells, and embryonic stem cells (70).

Another important miRNA in MS is miR.27a.3p, which is highly effective in distinguishing between different types of multiple sclerosis (RRMS, SPMS, and PPMS). It is associated with the T cell receptor signaling pathway and neurotrophin signaling pathway. miR-27a-3p targets several proteins involved in intracellular signaling networks that regulate the activity of mitogen-activated protein kinases (MAPKs) and nuclear factor-kB. It promotes the differentiation of Th17 and Th1 cells while increasing the accumulation of Tr1 and Treg cells. In MS patients, miR-27a-3p expression has been found to be increased in active brain lesions but decreased in the cerebrospinal fluid of patients with Alzheimer’s disease (71).

miR155 is essential for DC maturation and the ability of DCs to increase the activation of CD4+ T cell-specific antigens by inhibiting the expression of c-Fos, which can reduce the secretion of IL- 6, IFN-β, IL-12p70, TNF-α, and IL-12p40 through mature DCs. In mice with miR155 expression, the overexpression of Arg2 in DCs led to a severe depletion of arginine, which can prevent the reaction of T cells (72).

Overexpression of miR-132 in CD4^+^T cells in the EAE mouse model reduces the proliferation of IL-17 and IFN-γ secreting T cells. It has been observed that miR-26a decreased Th17 cell expression and increased Treg cell function by targeting IL-6. In EAE, inhibition of miR26a led to high levels of Th17 cytokines and exacerbation of EAE clinical symptoms. In contrast, Treg cell-specific transcription factors, Foxp3, were identified to be positively correlated with miR26a expression. Treatment of wild-type mice with anti-miR-21 oligonucleotide affected the disappearance of clinical EAE severity and the reduction of Th17 cells (73).

It has been reported that miR-20b, like a negative regulator of EAE, stops Th17 differentiation *in vitro* and showed that miR-20b is involved in the mechanisms of EAE through Th17. In addition, miR-30a, miR-326, and miR-23b interfere in the development of EAE by Th17 and through targeting Ets-1. Therefore, it seems that circulating miRNAs can be used as clinical biomarkers because they can reflect the severity and activity of the disease in MS patients. It has been reported that miR-155 is a new negative regulator of BBB function through modulating the regulator effects of the cell-to-brain endothelial cell (BEC) and cell-to-matrix, and it has led to impaired function of BBB in MS (73).

Overexpression of miR-125a-5p in brain endothelial cells reduces the expression of ICAM-1 and the migration of monocytes through brain endothelial cell barriers. Therefore, increasing miR-125a can cause normal function of the brain nerve fibers in neurological diseases induced by endothelial cells, especially in MS (74).

miR-214 increased in oligodendrocytes during differentiation and overexpression of miR-23a increases the differentiation of oligodendrocytes through reduction of lamin B. The increase in the level of miR-23a and miR-214 in MS lesions can reflect myelin regeneration (75). It has been reported that miR-124 is expressed in the M2 phenotype of macrophages and microglia because its overexpression results in the decrease in M1 markers such as TNF-α, IL-6, and iNOS and the increase in M2 phenotype-dependent proteins such as arginase-1, TGF-β, and FIZZI, which are necessary to stop EAE.

miR-181a is one of the first miRNAs that play an important role in the expansion and development of immune cells in thymus cells and has a low expression in the heart, bone marrow, and lymph nodes. The expression level of miR-181a is decreased in the expansion and differentiation of B cells from pro-B cells to the pre-B stage. For instance, miR-223 plays an essential role in the regulation and differentiation of granulocytic cells. Also, miR-150 is critical in differentiating B cells (76). Therefore, miRNAs play a vital role in differentiating different stages of immune cells.

Also, miR-155 plays an important role in the mammalian immune system, especially in regulating and differentiating T lymphocytes, germinal center reactions, and generating appropriate T cell-dependent responses. miR-155 can control mechanisms by regulating the production of cytokines. Many types of special Th cells, including Th9, Th17, Th1, Th2, Treg, and follicular helper T, differentiate in diverse pathways, but they can even have plasticity under certain conditions and become effector types. Recent studies have shown the role of miRNAs in the biogenesis of Treg and the prevention of autoimmune responses. Deficiency of miR-155 in Treg cells results in decreased expression of suppressor of cytokine signaling 1 (SOCS1) and activation of transcription factor (STAT5) in response to a limited amount of IL2. Forkhead box P3-(Foxp3-), dependent on miR-155 regulation, plays a role in Treg maintenance by targeting SOCS1 (77).

The production of B lymphocytes that express high-affinity receptors in the two stages of antigen-dependent selection in secondary lymphoid organs and selection in the bone marrow has been shown to be related to the dynamic setting out of miRNAs. In addition to antigenic selection, miRNAs play a role in expanding transcription factors and differentiation in the early stages of B cells. The continuous expression of miR-150, which is increased in the early stages of the stage of immature B cells and B cell differentiation, leads to the blocking of the expansion and development of B cells and the transition from pro-B to pre-B cells. miR-150 most likely reduces mRNAs that are involved in the activity and formation of pre- and pro-B cells (78).

### 3.3 Investigating the role of miRNAs in MS

In recent years, several studies have been conducted on the role of miRNAs in the development of MS, and these have shown that miRNAs are regulated and controlled by different pathways. Du et al. studied IL-17-producing Th17 cells and miR-326 expression in Th17 cells. Silencing miR-326 *in vivo* caused fewer Th17 cells and moderate EAE phenotype, while its overexpression caused more Th17 cells and aggravated EAE. This report shows the importance of the role of miRNA-326 in the differentiation of Th17 cells and the onset of MS (29).

Junker et al. discovered that several miRNAs, including miRNA-155, miRNA-34a, and miRNA-326, are present in active lesions of MS (28). They specifically identified astrocytes as a source of miR-155 expression and found dysregulated target sites for these miRNAs in the 3-UTR region of the CD47 transcript. Their model suggests that dysregulated miRNAs cause a decrease in CD47 levels, resulting in the loss of inhibitory control over brain cells and an increase in myelin sheath phagocytosis (28). Furthermore, Otaegui et al. discovered that miR-599 and miR-18b are associated with the regression state, while miR-96 is linked to remission (39).

Additionally, Zhang et al. found that certain miRNAs, including miR-22, miR-614, miR-1979, miR-648, miR-1826, miR-422a, and miR-572, exhibited different expression levels in the plasma samples of MS patients compared to the control group. These studies demonstrate that various biological samples contain miRNAs that have the potential to distinguish MS from other neurological diseases and differentiate between different types of MS (79).

Chapman et al. also demonstrated an increase in miR-17-5p in CD4+ cells of MS patients, which is associated with changes in the expression of its target genes such as phosphatase and PI3KR1. The relationship between miRNA expression and T-cell activation was supported by the elevated response of miR-17-5p in CD4+ cells *in vitro*. Other studies have provided new evidence regarding the role of miR-17-92 in autoimmunity (80).

Guraudi-Arellano et al. demonstrated that miRNAs such as miR-27b and miR-128 were elevated in miR-340 and T cells were present in the memory of CD4+ T cells in MS patients. These miRNAs played a role in the differentiation of pathogenic T cells. The altered expression of these miRNAs led to the differentiation of pathogenic Th1 cells in both human cells and mice suggesting their potential as therapeutic targets for regulating T-cell phenotypes in MS (81).

Additionally, Murugayan et al. found increased expression of miR-155 in CD4+ T cells from an EAE model. Mice lacking miR-155 showed reduced inflammation in the CNS and less severe disease symptoms. This study also revealed that miR-155 regulated Th1 cells producing IFN-γ, which contributes to inflammatory reactions in MS patients.

### 3.4 Serum miRNAs as biomarkers for diagnosis and treatment of MS

It is essential to develop novel, precise biomarkers that can clearly show the extent of disease improvement and treatment response in MS patients. MRI can identify plaques in the brain of MS patients, but these lesions may be related to other diseases although demyelination can be one of the systematic features of diseases such as lupus erythematosus, Behcet’s disease (BD), Sjogren’s syndrome (SS), Systemic Sclerosis tissue (SSc), or other very rare systemic autoimmune diseases. Some miRNAs showed a specific expression pattern in different diseases or disease stages. For example, Keller et al. have found that miR-145 is present in the blood of all MS patients.

In addition, Ridolfit et al. showed that miR-23a and miR-223 could play an essential role in the development of MS through the NF-kb pathway and FGF gene. They also found that rs1044165 SNP of miR-223 acts as a protective factor, and rs3745453 SNP of miR-23a is a risk factor for MS (80).

Recently, miRNAs have been considered as particular targets for treating autoimmune diseases. Murugaiyan et al. showed that inhibiting miR-21 by locked nucleic acid (LNA)-anti-miR decreases the expression of cytokines in TH17 cells, especially IL-17F, IL-17A, IL-21, and IL-22, and it can effectively prevent disease progression in the EAE model. They also reported that the expression of miR-21 increased in miR-21, and TH17 cell expression-deficient mice are resistant to EAE (82).

Guinea-Viniegra et al. reported that using antagonizing miR-21 improved psoriasis in psoriasis-like mouse and mouse xenotransplantation models. Further studies showed that miR-21 promotes TH17 responses through SMAD-7, a negative regulator of the TGF-β signaling pathway. Also, miR-21 increases the activity of SMAD-2/3, suppresses the expression of IL-2, expands the TGF-β signaling pathway, and finally increases TH17 differentiation (83).

miRNA-based therapies can include the use of specific miRNA mimics to reduce target gene expression or the use of antisense oligonucleotides to block specific proteins. In particular, the anti-miRNA therapeutic strategy is preferred to the antisense mRNA therapeutic strategy because a miRNA affects clusters of genes involved in a disease. A promising strategy is using LNAs (locked nucleic acids), a bicyclic RNA conformational analog with a high affinity to its complementary RNA molecule and remarkable stability in blood and tissue *in vivo*.

Recent *in vivo* studies on the potential application of LNA-modified oligonucleotides on the function of miRNAs have led to the expansion of their use for therapeutic purposes. The LNA-modified miR-122 inhibitor was used for treating hepatitis C infection in phase one clinical trials (84, 85). Recent studies have shown that miR-340, miR-128, and miR-27b increase CD4+ T cells of MS patients and cause a shift in the production of TH2 to TH1 cytokines. *In vitro*, using miRNA inhibitory oligonucleotides, including miR-340, miR-27b, and miR-128, has increased TH2 responses. Therefore, the results indicate that the inhibition of these miRNAs *in vivo* can be used as a therapeutic target for MS disease (86).

### 3.5 Profile changes of miRNAs in the brain

**Figure 1:**
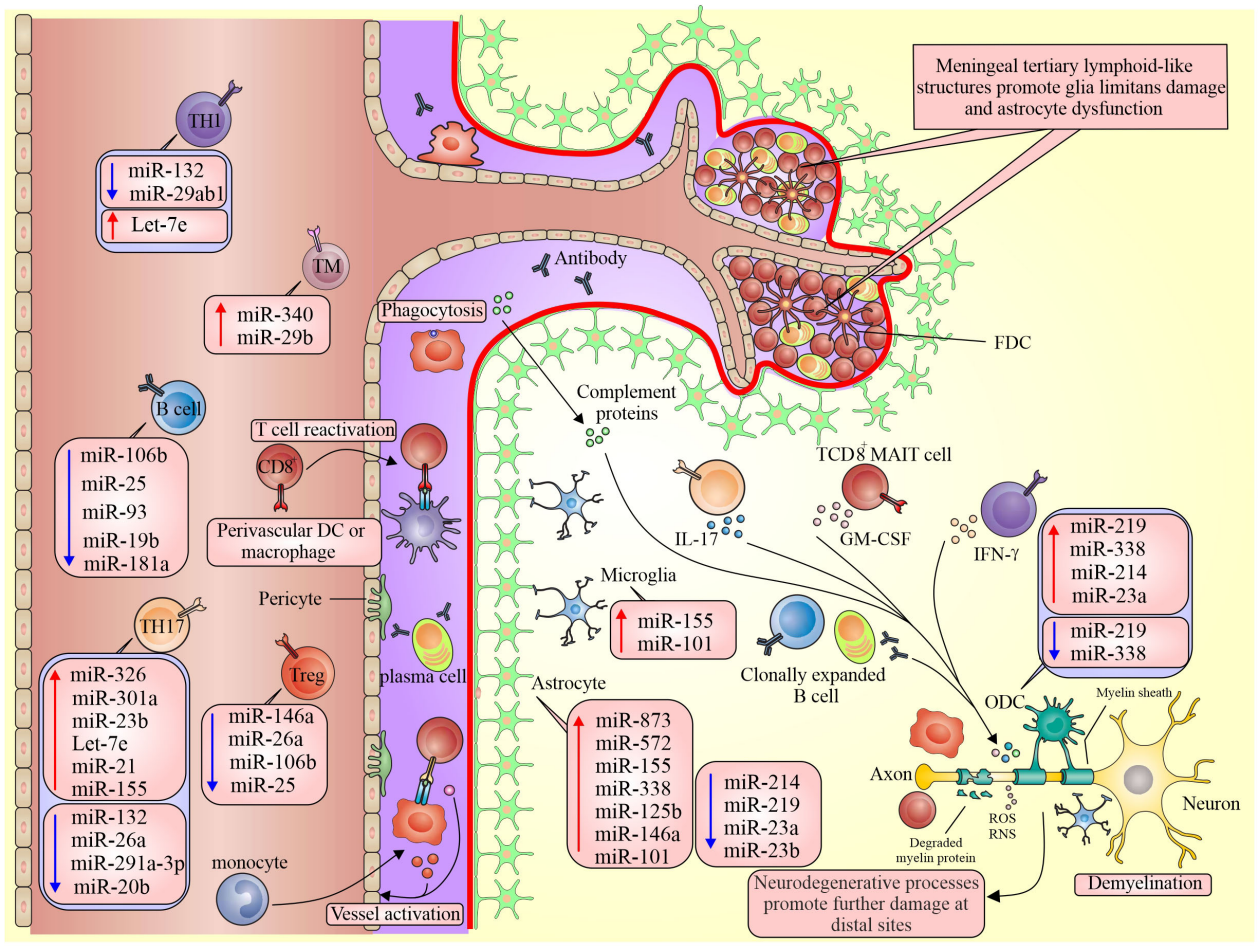
Different expressions of miRNA in pathological processes of MS. Dysregulation of miRNAs in active lesions alters the BBB, compromising the brain’s immune privilege. This results in BBB dysfunction and the exposure of neuronal antigens to the peripheral immune system, leading to inflammation. Subsequently, this provokes the release of pro-inflammatory cytokines, further exacerbating the inflammatory response in the brain and contributing to CNS injury.

Changing the pattern of miRNAs can reflect both the demyelination process and the attempt to regenerate degenerated axon myelin in the CNS. It is controversial whether a disrupted expression of miRNAs leads to MS. The disruption of miRNAs may play an essential role in many biological progressions such as reproduction, differentiation, development, metabolism, angiogenesis, inflammation, immunity, and apoptosis (**Figure 1**).

Fenoglio et al. have shown significantly increased expression levels of miR-150, miR-21, miR-155, and miR-146a in RRMS patients compared to healthy control subjects (63). Statistical investigations and analyses on real-time PCR showed that miRNA-142-3p, miRNA-21, miRNA-146b, miRNA-146a, and miRNA-155 are increased in spinal cords in the EAE mouse model (63). Also, the expression of miRNA-155 was significantly increased in the lymph nodes, spleen, and brain of EAE mice (63).

In particular, Junker et al. have shown that miR-155, miR-34a, and miR-326 have increased expression in active lesions of MS patients compared to healthy individuals. The increased expression of these miRNAs in active MS lesions leads to decreased CD47 expression and failed inhibitory control of macrophages, resulting in myelin phagocytosis (28, 87). Analysis of the researchers’ data showed that miR-142-3p, miR-21, miR-146a, miR-326, miR-155, and miR-146b increased in the brain white matter of MS patients with MS and EAE mice (88).

### 3.6 Extracellular profile of microRNAs

Investigators have conducted microarray analysis on more than 900 miRNA transcripts collected from plasma samples of MS patients. Six of 900 miRNAs (miR-614, miR-648, miR-572, miR-1826, miR-22, and miR-422a) significantly increased, and miR-1979 significantly decreased in MS patients. It has been shown that miR-223 plays an essential role in granulopoiesis by targeting mef-2c and modulating the NF-κb pathway associated with modulating immune inflammatory responses (46).

The results revealed that miR-145 has considerably increased expression in PBMC of MS patients, and expression of miR-7-1-3p, miR-20a-5p, miR-15b, miR-223, and miR-23a is decreased in the blood of MS patients. miR-142-3p, miR-21, miR-146a, miR-146b, miR-326, and miR-155 expression significantly increased in PBMC and white matter of brain lesions of MS patients compared to healthy controls (26, 89), which is suggested to be used as a diagnostic and differential marker for MS in patients.

## 4 The biological mechanisms of the BBB

The presence of the BBB creates a controlled microenvironment by regulating the exchange of ions and molecules between the bloodstream and brain tissue (90). Several investigations have uncovered the physiological functions of the BBB, which include protecting the brain. In addition to physically preventing potentially harmful substances from entering, the BBB also serves multiple roles in maintaining homeostasis, facilitating the transport of essential molecules, and regulating inflammation (91, 92). The BBB sustains brain homeostasis by controlling specific ion channels and transporters (93, 94).

As a highly dynamic and intricate structure, the BBB consists of pericytes, astrocytes, endothelial cells, and the basement membrane. It modulates the movement of substances between the central nervous system and the bloodstream (94, 95). It is known that the function of the BBB is tightly regulated by specific tight junction proteins. Notably, claudin-5, the most prominently expressed tight junction protein at the BBB, is closely associated with the pathology of ischemic stroke (96).

Previous studies have reported that many miRNAs are now thought of as MS biomarkers due to the pathology of MS being related to the abnormal expression of miRNAs in CNS lesions. To develop effective strategies for promoting remyelination, it is imperative to comprehend the role that miRNAs play in the regulation of oligodendrocyte development and myelination (2, 97).

A comprehensive understanding of the advantages and disadvantages of various strategies aimed at overcoming the challenges associated with efficient miRNA delivery is critical in the development of safe and efficacious therapeutic approaches for preserving BBB integrity in a variety of CNS diseases (2, 98).

The previous findings confirm that the development of novel therapeutic approaches, specifically targeting brain parenchyma, is imperative, as most existing therapies struggle to efficiently penetrate the blood-brain barrier (BBB). The preservation of BBB’s integrity is vital for maintaining brain homeostasis and normal neurological function (2, 98).

### 4.1 microRNAs and their impact on BBB tight junctions: an examination of molecular mechanisms and therapeutic prospects

In the context of MS and related diseases, the endothelial barrier is compromised, contributing to the development and progression of these conditions (99).

Numerous investigations have primarily concentrated on the pivotal tight junction components of the BBB, specifically claudins, ZO, and occludin proteins. A reduction in the expression of ZO-1 and claudin-5 within brain endothelial cells has been associated with several neurological disorders (75, 99). Claudin-5, as the most prominently expressed tight junction protein in the BBB, exhibits a close association with ischemic stroke pathology (96).

Recent research has recognized the direct targeting of these junction molecules by microRNAs, leading to alterations in BBB integrity (74). These findings strongly indicate that the repair of tight junctions may serve as a therapeutic target for restoring BBB integrity and improving disease management. To date, there are no known molecules capable of directly enhancing the expression of tight junction components to restore BBB integrity (100).

Previous studies have reported that *in vivo* administration of locked nucleic acid-modified antisense oligonucleotides, targeting miR-501-3p, resulted in the restoration of ZO-1 gene expression and amelioration of BBB disorder in mice afflicted with vascular cognitive impairment, leading to a significant improvement in cognitive function. The BBB, a highly intricate and dynamic structure, rigorously regulates substance exchange through the complex interplay of tight junction proteins among endothelial cells (96, 101).

Utilizing in silico analysis and subsequent confirmatory laboratory experiments, several miRNAs have been identified as regulators of specific diseases, with some of them being related to tight junctions. A fundamental feature of the BBB is the presence of exceptionally robust tight junctions between brain endothelial cells, controlled by an array of junction molecules, covering adherens junction proteins and tight junction (96).

Remarkably, a notable reduction in miR-125a-5p expression was observed in the inflamed blood vessels of multiple sclerosis patients compared to non-inflamed blood vessels, suggesting a positive correlation between BBB integrity and miR-125a-5p during inflammation (74). Upon treatment of brain endothelial cells with the proinflammatory mediator tumor necrosis factor-alpha/interferon-gamma, a significant alteration was noted in 107 miRNAs, including the downregulation of miR-125a-5p (74, 102). Conversely, miR-155 was found to negatively impact BBB integrity during inflammation (102). miR-101 indirectly downregulates claudin-5, ultimately increasing BBB permeability (103). miR-373, miR-27a, and miR-210 are involved in modulating the integrity of the BBB by affecting proteins such as VE-cadherin, occludin, and ZO-1 in various brain disorders (99).

miR-96 indirectly suppresses ZO-1 mRNA expression, thereby promoting monocyte migration across the BBB (104). Also, miR-424-5p binds to the 3’ untranslated region of endophilin-1 mRNA, upregulating endophilin-1 mRNA levels (103, 105). The overexpression of endophilin-1 leads to the inhibition of the EGFR-Jun-N-terminal kinase and EGFR-ERK signaling pathways, resulting in the downregulation of occludin proteins and ZO-1 in vascular endothelial cells (103, 105).

Abundant miRNAs have been identified as direct or indirect regulators of specific barrier proteins. In a prior study, it was demonstrated that miR-27a targets VE-cadherin to disrupt vascular integrity (106). Consequently, a potential therapeutic approach to enhance VE-cadherin expression may thereby resolve abnormal vascular integrity (107). Therefore, miR-27a emerges as a potential common regulator of vascular integrity, particularly within the cerebral vascular system (106, 107).

The most critical tight junction (TJ) proteins linked to the BBB include claudin-5, occludin, and ZO-1 (108). miR-96 indirectly inhibits the expression of ZO-1 mRNA, leading to increased migration of monocytes across the BBB (104). MiR-424-5p binds to the 3’ untranslated region (UTR) of endophilin-1 mRNA and upregulates endophilin-1 mRNA (103, 105). Overexpression of endophilin-1 results in the inhibition of the EGFR-Jun-N-terminal kinase and EGFR-ERK signaling pathways, subsequently downregulating occludin proteins and ZO-1 in vascular endothelial cells (103, 105). Also, miR-101 indirectly downregulates claudin-5, resulting in increased BBB permeability (103).

The loss of myelin is a pathological hallmark in MS, and various factors influence the processes of developmental myelination and remyelination within the central nervous system (CNS). Many of these factors impact the exceedingly regulated transition from an immature, proliferating oligodendrocyte progenitor cell (OPC) to a mature, functional, myelinating oligodendrocyte (OL) (75).

microRNAs upregulated in active MS lesions, including miR-326, miR-155, and miR-34a target the 3’-untranslated region of CD47 in reporter assays. This targeting reduces CD47 levels in brain resident cells, shifting macrophages from inhibitory control (28). Furthermore, miR-23b has been found to suppress auto-inflammatory responses associated with IL-17 in systemic lupus erythematosus (SLE) and mouse models of MS (109).

In individuals with MS, there is a notable reduction in the levels of miR-223, miR-15b, and miR-23a in their serum (110). Specifically, miR-23a/b can be regarded as a distinctive astrocytic marker, and its expression is influenced by stimuli promoting astrocyte differentiation (111). miR-23a, linked with certain long non-coding RNAs (lncRNAs), participates in a complex regulatory network that modulates critical genes for myelin formation and maintenance. miR-23a appears to play a role in advancing less-differentiated oligodendrocyte progenitor cells (OPCs) toward myelination and mature oligodendrocytes (OLs), likely by enhancing the expression of genes enriched in mature OLs. This regulatory mechanism may also comprise the suppression of genes enriched in neurons and, to a lesser extent, astrocytes (112, 112). Astrocytes, a type of glial cell in the central nervous system, assume diverse responsibilities in maintaining a healthy neural environment and the integrity of the BBB (113).

The elevated levels of miR-23a, a microRNA that promotes myelin production, in MS brain tissue may be attributed to the ongoing remyelination process in MS through OLs. However, it is worth noting that peripheral immune cells in MS patients also exhibit increased miR-23a expression. Therefore, the heightened miR-23a levels in the MS brain may stem from increased infiltration of immune cells (112). Taken together, the contrasting regulation of miR-23a in OPCs and astrocytes suggests a complex interplay of molecular events during MS. Future studies on the current issue are therefore required to comprehensively understand the specific targets and functions of miR-23a in these cell types in the context of MS.

### 4.2 Neuroinflammation and the BBB

Under normal physiological conditions, there is a minimal level of immune cell trafficking across the BBB as a component of the brain’s immunosurveillance, which is essential for effective immune response within the brain and clearance of infectious pathogens (114, 115). In the presence of inflammation, a disrupted BBB compromises the brain’s immune privilege, exposing neuronal antigens to peripheral inflammatory molecules. This exposure further stimulates the inflammatory response within the brain, ultimately accelerating the development of neurological diseases (2).

Inflammatory cytokines, including interleukin-1β, TGF-β, TNF-α, and IL-6, have been shown to be upregulated during pathological processes that compromise BBB function (2, 116, 117). Several lines of evidence from animal studies also support the role of IL-1β in BBB dysfunction during neuroinflammation. Treatment with IL-1β receptor antagonists or genetic deletion of the IL-1 receptor attenuates neuroinflammation-induced BBB hyperpermeability in mice (2, 118).

microRNAs can also utilize their influence on markers or inflammatory cytokines in order to regulate the integrity of the BBB. For instance, miR-21-5p modulated the BBB through the targeting of pro-inflammatory cytokines such as TNF-α, NF-kB, and IL-6 signaling pathway (119). Furthermore, the enhanced expression of let-7g and miR-98 has been found to diminish neuro-inflammation prompted by BBB permeability (120).

Moreover, it has been reported that several miRNAs such as miR-125-5p and miR-320a have shown reduced expression in brain/spinal cord tissues or blood samples from patients with multiple sclerosis, as well as in *in vitro* BBB models exposed to pro-inflammatory cytokine. These downregulated miRNAs contain miR-126, miR-125-5p, miR-126, and miR-320a as reported in various studies (121, 122).

In cases of brain tumors, regions with an intact BBB hinder the delivery of drugs to tumor cells at therapeutic concentrations. In the context of BBB penetration, exosomes can transport endogenous peptides and proteins from one side of the BBB to the other. This indicates that the paracellular pathway is less critical than previously believed for delivering therapeutics to tumors (123). Exosomes are extracellular vesicles and small membrane-bound carriers released by cells to facilitate communication with distant or neighboring cells. Exosomes possess unique properties, including specific surface proteins, which guide them to specific recipient cells for binding and uptake (124, 125).

Remarkably, exosomes have emerged as potential contributors to the preservation of BBB integrity, such as exosomes derived from neurons that carry miR-132. These specialized exosomes facilitate communication between brain endothelial cells and neurons, ultimately playing a significant role in upholding the integrity of the BBB (2).

To ensure inherent compatibility with human cells, extracellular vesicles (EVs) efficiently transport cellular membranes and do not encounter various challenges related to drug delivery, including issues like RNA degradation, accumulation in endosomes, susceptibility to phagocytosis, resistance to multiple drugs, potential cytotoxicity, and immunogenic responses (126, 127). It is recommended that further research should be undertaken using the following strategies and utilizing exosomes for miRNA and mRNA delivery in the treatment of various diseases such as MS that has been provided (127).

## 5 Associated miRNA alteration of MS in response to treatment

Some recent studies have investigated the effect of some usual drugs used in MS on miRNA levels. Ingwersen et al. found that miR-29a, miR-103, miR-18, and miR-20b were increased after natalizumab treatment, and miR-20b and miR-326 play an essential role in regulating Th17 responses and blood-brain barrier damage in patients. miR-29a was also involved in the differentiation and expansion of Th1 responses. Petrocca et al. reported that natalizumab increased miR-106b and decreased the expression of miR-17, which are miRNAs involved in TGF-𝛽 signaling and the expansion of CD4+ T cell responses by targeting TGFBR2.

Also, the reduction of miR-17 expression has been associated with the elevation of pro-apoptotic proteins, including the BIM, Bcl-2 family, the cyclin-dependent kinase inhibitor 1, p21, and E2F1. These targets, which are altered by natalizumab, can modify cellular responses in the EAE model and correlate with disease severity inversely. Therefore, the effect of natalizumab on miRNA expression can help to comprehend its impact on the process of pathogenesis and disease progression. It has been shown that INF-𝛽 treatment increased the expression of miR-26a-5p in MS patients. It has been reported that miR-26a-5p is associated with the reduction of postsynaptic density protein 95 (DLG4), which plays a crucial role in neuron signaling after 3 months of INF-𝛽 treatment (128).

This evidence suggests that these markers may be used as novel biomarkers to investigate the therapeutic effect of IFN-𝛽 and develop new treatments for MS patients. Treatment with Glatiramer acetate (GA) can reduce miR-142-3p and miR-146a expression in PBMC of MS patients, in which miR-142-3p can affect the expansion of Treg responses. Reducing miR-146a expression can shift Th1 responses to Th2 and improve MS patients by inhibiting monocyte response (129) (**Table 2**). Understanding the complexity of the miRNA network can open a new window for finding individual biomarkers in clinical diagnosis and monitoring the effectiveness of treatment. These findings showed that miRNAs may support and evaluate therapeutic effects.

**Table 2:** MiRNA biomarkers of therapeutic response in multiple sclerosis (MS).

Drug treatment	Increased miRNA	Decreased miRNA	Reference
NatalizumAb	miR-18a, miR-20b, miR-29a, miR-103 , miR-26a, miR-155	miR-326, miR-17 , miR-155 and miR-26a	(Ingwersen et al., 2014) (130) (Mameli et al., 2016) (131)
INF-*β*	miR-26-5p, miR-16-5p, miR-342-5p, miR-346, miR-518b, miR-760, let-7a-5p, and let-7b-5p	miR-27a-5p, miR-29a-3p, miR-29b-1-5p, miR-29c-3p, miR-95, miR-149-5p, miR-181c-3p, miR-193a-3p, miR-193-5p, miR-423-5p, miR-532-5p, miR-708-5p, and miR-874	(Hecker M et al,2013) (9) (De Felice B al,2014) (132)
Glutiramer Acetate	–	miR-146a, miR-142-3p	(Waschbisch et al.,2011) (133)
GSK3β*β*inhibitors	–	miR-98 and let-7g	(Rom S et al,2015)(120)
Dimethyl fumarate	–	miR -146a and -155 , miR-125a	(Giuliani et al., 2021) (134)
Fingolimod	–	miR-15b, miR-23a, and miR-223	(Fenoglio et al., 2016) (135)

### 5.1 miRNAs as molecular medicine

The development of disease-modifying drugs for the treatment of MS has recently received much attention. Interferon-β (IFN-β) and glatiramer acetate reduce the risk of patients returning to the level of RR-MS; approximately one-third of RR-MS patients had an adverse (inadequate) response to drugs. It is one of the important principles that have been the focus of researchers who use miRNAs as new molecular drugs for MS. However, similar problems have been observed with other drugs, such as toxicity, side effects, and stability of miRNAs in target cells, which must all be resolved.

Also, miRNA selection as a drug for a pathological condition should be based on target detection and confirmation. The role of genes in disease is another factor in adopting miRNA as a drug. Vandenbroek et al. discovered that SOCS1 is a crucial regulator of cytokinin signaling. It has been identified that IFN-β performed modulatory effects on the immune system through the induction of SOCS3 and SOCS1 in various types of inflammatory sub-cells, thereby preventing inflammatory reactions in cells. It has been reported that SOCS1 is a therapeutic tool for the EAE mouse model of multiple sclerosis. Therefore, SOCS1-targeting miRNAs may be good candidates for influencing immune responses to cytokinins by regulating SOCS1 expression.

Although no miRNA drug candidates have reached phase 3 trials according to the “Clintrials.gov” database, there are ongoing phase trials for drugs like miR-122/Miravirsen and MRX34. miR-122/Miravirsen is an anti-miR drug composed of locked nucleic acid (LNAs) ribonucleotides that are currently being tested for the treatment of HCV infections (130). MRX34 is a liposomal formulation of miR-34a, which is a tumor suppressor that is often lost or expressed at reduced levels in various types of tumors. It is being investigated as a potential first-in-class miRNA mimic cancer therapy (131, 132). MRX34 has been registered in the “Clintrials.gov” database for a Phase 1 study on microRNA-based cancer therapy (133).

A major challenge lies in delivering drugs to the brain. Various methods such as modified micelles, liposomes, nanoparticles, intranasal administration, and other delivery approaches have been tested to overcome the BBB with varying degrees of success (134). One complex approach involves designing lipid-soluble small molecules weighing less than 400 daltons that can successfully cross the blood-brain barrier, while macromolecules struggle to penetrate it effectively. miRNA-based drugs have demonstrated significant effectiveness in treating various health conditions such as hepatitis C, cancer, cardiac abnormalities, and pathological fibrosis kidney disease. Some miRNA candidates have shown promising results in clinical trials, while others have not been successful. Additionally, there is an ongoing Phase 1 trial for MRG 110, an antisense oligonucleotide that uses locked nucleic acid (LNA) to block the function of miR-92 (135). MiRagen has recently announced a Phase 1 trial for MRG 110 as a potential application in wound healing and heart failure (135, 136). MiRagen also has active Phase 1 and Phase 2 trials for miR-155 (Cobomarsen; MRG-106) and miR-29 (MRG-201), respectively, targeting keloid and scar tissue formation and T lymphoma patients. In 2019, Regulus suggested a new potential miRNA drug called RGLS5579, which targets miR-10b and may be used in trials for patients with glioblastoma multiforme (137). Recently, a Phase 1 trial using a newer technology called “TargomiR” showed promising results in patients with non-small cell lung cancer or recurrent malignant pleural mesothelioma (**Table 3**).

**Table 3:** Interventional clinical trials for miRNAs.

miRNA gene; drug name	Clinical trial number; phase status	Disease/disorder investigated
**miR-34; MRX34**	NCT01829971; phase 1 (terminated)	Liver cancer, lymphoma
	NCT02862145; phase 1 (withdrawn)	melanoma
**miR-92; MRG 110**	NCT03603431; phase 1 (recruiting)	wound healing, heart failure
**miR-16;MesomiR-1**	NCT02369198; phase 1 (completed)	Mesothelioma, lung cancer
**miR-122; Miravirsen**	NCT02508090; phase 2 (completed)	Hepatitis C virus
	NCT02452814; phase 2 (completed)	
	NCT01200420; phase 2 (completed)	
	NCT01872936; phase 2 (unknown status)	
	NCT01727934; phase 2 (unknown status)	
	NCT01646489; phase 1 (completed)	
**miR-29; MRG-201**	NCT03601052; phase 1 (recruiting)	Keloid, fibrous scar tissue formation
**miR-21; RG-012**	NCT02855268; phase 2 (suspended, sponsor decision)	Alport syndrome
	NCT03373786; phase 1 (active, not recruiting)	
**miR-155; Cobomarsen (MRG-106)**	NCT03713320/phase 2 (recruiting)	T-cell lymphoma/mycosis fungoides
	NCT03837457/phase 2 (new/not yet recruiting)	

Briefly, TargomiR delivery vehicles contain a miRNA mimic, a small cell derived from bacteria, and a targeting component such as an antibody that recognizes a specific protein in the target cells. In the first human trial of the drug TargomiR called MesomiR-1, the miRNA mimic miR-16 was found to be a tumor suppressor and the target group was lung cancer cells that overexpressed the EGFR antibody (138, 139). These studies provide hope for mesothelioma patients who have low survival rates (136). The first human trial of siRNA took place in 2004 and the first siRNA drug was approved in 2018 (140).

## 6 Conclusion

miRNAs play roles in various cellular processes and have been identified as post-transcriptional gene expression regulators. miRNAs play a key role in various vital processes such as cell growth, development, apoptosis, and cell differentiation. miRNAs’ function and regulation of their expression are still largely unknown, and even more complex is the regulatory network between miRNAs and their target genes. Recent studies have revealed a unique miRNA pattern in different body tissues. To date, clinical applications in peripheral tissues have been reported for miRNA mimics (to overexpress transcripts) and miRNA suppressors (to silence the function of transcripts). Dysregulated miRNAs influence the inflammatory response and BBB permeability. miRNA alterations can serve as potential biomarkers to assess treatment efficacy. The altered expression of specific miRNAs has been linked to the development of MS and the differentiation of pathogenic T cells. Furthermore, miRNAs have emerged as promising candidates for distinguishing different types of MS and assessing treatment responses.

As discoveries from the Human Genome Project continue to shape clinical research, emerging miRNA drugs offer potential treatments for health conditions that cannot be addressed by current options. Bioinformatics programs aid in related biological pathways and identifying miRNA binding sites in target genes. *In vivo* experiments have also played a role in accelerating the translation of miRNAs into clinical medicine. These experiments have paved the way for investigating miRNAs, which are non-coding RNA transcripts that outnumber protein-coding genes. Future human experiments will likely focus on epigenetic targets. MiRNAs could serve as valuable diagnostic tools as their expression patterns in biological samples, such as blood and cerebrospinal fluid, can differentiate MS from other neurological diseases. Additionally, they discuss the challenges of drug delivery to the brain and the potential of miRNA-based therapies, including miRNA mimics and antisense oligonucleotides, as a novel approach to treating MS by regulating the expression of specific target genes. Ongoing clinical trials are investigating the potential of miRNA-based therapies in various diseases, including cancer and MS. Research into miRNA-based therapies is ongoing, offering potential avenues for the development of novel treatments for MS and other diseases. Understanding the complex network of miRNAs in MS provides valuable insights into the disease’s mechanisms and potential therapeutic interventions.

## Author contributions

AEN: Conceptualization, Data curation, Writing – original draft. SMMZ: Writing – review & editing. HN: Writing – review & editing. ASA: Methodology, Writing – review & editing. AF: Investigation, Methodology, Writing – review & editing. MA: Investigation, Writing – review & editing. RN: Conceptualization, Writing – review & editing. SS: Writing – review & editing. MM: Conceptualization, Methodology, Supervision, Writing – review & editing.
